# Characterization and Validation of the Antibacterial Activity of *Heyndrickxia coagulans* BHE26 Against *Helicobacter pylori*

**DOI:** 10.3390/foods15010131

**Published:** 2026-01-01

**Authors:** Nannan Wang, Changhe Ding, Jun Gao, Lingguang Du, Dongge Zheng, Zhihui Hao, Zhuoran Ren, Haiwei Lou

**Affiliations:** 1 College of Food Science and Engineering, Henan University of Technology, Zhengzhou 450001, China; 2 Food Laboratory of Zhongyuan, Luohe 462300, China; 3 Zhengzhou Jinbaihe Biological Engineering Co., Ltd., Zhengzhou 450001, China; 4 College of International Education, Henan University of Technology, Zhengzhou 450001, China

**Keywords:** *Heyndrickxia coagulans* BHE26, probiotic, anti-*Helicobacter pylori*, gastric tissue microbiota

## Abstract

*Helicobacter pylori* infection is a primary cause of gastritis and gastric ulcers. It is crucial to find alternative therapies for *H. pylori* infection due to the significant side effects of current antibiotics. *Heyndrickxia coagulans* is an ideal probiotic due to its functionality and stability in production and storage. This study explored the anti-bacterial effects of *H. coagulans* BHE26 in vitro and in vivo. *H. coagulans* BHE26 showed notable tolerance to simulated gastric juice (pH 3.0) and 1% bile salts, highlighting its potential suitability for gastrointestinal survival. *H. coagulans* BHE26 was resistant to ceftriaxone but sensitive to penicillin, ampicillin, erythromycin, gentamicin, ciprofloxacin, ceftriaxone, lincomycin, tetracycline and chloramphenicol. These characteristics showed that *H. coagulans* BHE26 is a potential probiotic bacterium. In vitro assays demonstrated that *H. coagulans* BHE26 inhibited *H. pylori*, reduced urease activity, and displayed notable auto-aggregation and co-aggregation abilities. In vivo, administration of *H. coagulans* BHE26 alleviated *H. pylori*-induced gastric mucosal damage, significantly lowered serum anti-bacterial IgG levels, and modulated gastric microbiota composition, including an increase in *Turicibacter* and a decrease in *Lactobacillus* abundance. These results indicate that *H. coagulans* BHE26 alleviated *H. pylori*-induced inflammation, offering a novel therapeutic strategy against *H. pylori* infection.

## 1. Introduction

*Helicobacter pylori* is a widespread human pathogen that colonizes the gastric mucosa [1,2]. Over half of the global population is infected with *H. pylori*, with a reported prevalence of 55.8% in China [3]. Long-term infection can cause a series of gastrointestinal diseases, such as chronic gastritis, peptic ulcers, gastric cancer, and gastric mucosa-associated lymphoid tissue lymphoma [4,5]. *H. pylori* infection and its related diseases pose a substantial health burden in China and remain a significant global public health concern [6]. Antibiotic-based regimens, primarily comprising amoxicillin and metronidazole, remain the first-line treatment for *H. pylori* infection [7]. In recent years, antibiotic resistance has become an increasing concern worldwide. Rising antibiotic resistance has steadily reduced the success of *H. pylori* eradication [8]. Additionally, antibiotic treatment can disrupt gut microbiota, causing adverse effects such as diarrhea, anorexia, and bloating [9,10]. These risks are particularly concerning for vulnerable populations, including children, pregnant women, and lactating individuals [11]. Thus, identifying safer and more effective eradication strategies remains a major global clinical challenge.

Probiotics, defined as live microorganisms that confer health benefits, are widely used to maintain the microbial homeostasis and support intestinal health. In *H. pylori* eradication programs, probiotics have gained increasing attention for their ability to enhance eradication rates and reduce the adverse effects of antibiotic therapy [12,13,14]. Both the European Maastricht VI Consensus and the Sixth Chinese Consensus on *H. pylori* infection recommend probiotic supplementation as an adjunct to standard eradication regimens [15,16,17]. Thus, identifying probiotics with strong anti-bacterial activity is crucial for improving treatment strategies.

*Heyndrickxia coagulans* is a thermotolerant, spore-forming, lactic acid-producing bacterium. Its ability to form resistant endospores ensures high survival during storage, gastrointestinal transit, and food processing [18]. In recent years, the growing consumer demand for health-promoting foods has driven a rapid expansion in the application of probiotics in the functional food industry. *Heyndrickxia coagulans* is used in the food industry, such as in wine making and sugar production, owing to its butyric acid-producing and fermentation capabilities [19,20]. It is used as a food additive for dairy products and pasta due to its excellent heat resistance and harsh-environment resistance [20]. Notably, *H. coagulans* is the only spore-forming bacterium approved for use as a food ingredient in China, offering advantages in storage and transportation, and application [21,22]. However, studies on its inhibitory effects against *H. pylori* are still limited. Current research on *H. coagulans* primarily focuses on its role in modulating immunity, alleviating constipation, mitigating acute alcohol intoxication and restoring intestinal microbiome homeostasis [23,24,25]. Fermented foods represent rich and diverse microbial ecosystems that have yielded multiple *Lactobacilli* with anti-bacterial activity [26]. However, *H. coagulans* with comparable inhibitory potential remains largely underexplored. To date, no systematic studies have isolated and characterized *H. coagulans* from fermented foods with anti-*H. pylori* activity. Therefore, screening *H. coagulans* with anti-*H. pylori* activity from fermented foods (homemade soybean paste) is crucial for the prevention and treatment of *H. pylori* infections.

We have isolated a strain of *H. coagulans* BHE26 with inhibitory effects against *H. pylori*, deposited under the accession number CGMCC No. 30414 [27]. The inhibitory and strain-specific properties were analyzed after determining anti-bacterial activity, acid resistance, hydrophobicity, auto-aggregation and co-aggregation. Animal experiments evaluated the effects of *H. coagulans* BHE26 on *H. pylori* infection in vivo, using gastric urease activity, histopathology, serum anti-bacterial IgG levels, and microbiota changes as key indicators. This study developed a *H. coagulans* strain with gastric tolerance and anti-bacterial activity, suggesting its potential application in dietary interventions for *H. pylori* management.

## 2. Materials and Methods

### 2.1. Bacterial Strains and Culture Conditions

*H. coagulans* BHE26 (CGMCC No.30414, E26) was stored at the China General Microbiological Culture Collection Center (CGMCC). *H. pylori* SS1 (GDMCC 1.1824) was obtained from Hangzhou Baosai Biotechnology Co., Ltd. (Hangzhou, China). *Lactobacillus plantarum* CN2018 (LP) was provided by Hebei Yiran Biotechnology Co., Ltd. (Shijiazhuang, China). Brain heart infusion broth (BHI) was from Hope Bio-Technology Co., Ltd. (Qingdao, China). The bile salts and urease were from Beijing Solarbio Science and Technology Co., Ltd. (Beijing, China). Fetal bovine serum (FBS) was obtained from Yuanye Bio-Technology Co., Ltd. (Shanghai, China). Pepsin, trypsin, proteinase K, papain, and bile salts were obtained from Sigma-Aldrich (St. Louis, MO, USA). The other reagents were of analytical grade.

*H. pylori* was cultured on Columbia agar Base (CAB) under microaerophilic conditions for 72 h. *H. pylori* colonies were scraped from the agar surface and resuspended in BHI broth supplemented with 10% fetal bovine serum to adjust the bacterial concentration to an optical density (OD_600_) of 0.10 ± 0.01. The modified glucose-supplemented de Man Rogosa Sharpe (GMRS) agar (20 g glucose; 10 g yeast extract; 10 g peptone; 2.68 g K_2_HPO_4_·3H_2_O; 0.05 g manganese sulfate; 0.048 g magnesium sulfate; 2 g ammonium citrate; and 15 g agar dissolved in 1 L distilled water).

### 2.2. In Vitro Anti-Bacterial Activity of H. coagulans BHE26

The anti-bacterial activity of *H. coagulans* BHE26 was evaluated using the agar well diffusion assay as described by Paucar-Carrión et al. with minor modifications [28]. Briefly, *H. pylori* suspension (OD_600_ = 1.0 in BHI) was spread evenly on Columbia blood agar plates supplemented with 10% fetal bovine serum. Wells with a diameter of 6 mm were made using a sterile cork borer. Each well was filled with 50 μL of GMRS (negative control), *Lactobacillus plantarum* CN2018 (positive control), 50 μL of phosphate-buffered saline (PBS) containing *H. coagulans* BHE26 (1.5 × 10^8^ CFU/mL), or 50 μL of the supernatant obtained from *H. coagulans* BHE26 culture after centrifugation at 8000× *g* for 10 min using a high-speed refrigerated centrifuge (LYNX 6000, Thermo Fisher Scientific, Waltham, MA, USA). The plates were incubated under microaerophilic conditions at 37 °C for 72 h, and the diameters of the inhibition zones were measured. The values were determined by three independent experiments.

### 2.3. Characterization of Antimicrobial Compounds from H. coagulans BHE26

Protease sensitivity assays: The cell-free supernatant (CFS) from *H. coagulans* BHE26 was treated with different enzymes to determine the nature of the compounds responsible for the antimicrobial activity. Pepsin (pH 3.0), acid protease (pH 3.0), trypsin (pH 7.0), proteinase K (pH 7.0), or papain (pH 7.0) was added to 50 mL CFS to a final concentration of 1 mg/mL at 37 °C for 1 h. To evaluate the influence of pH, the CFS was adjusted to pH 7.0 using 1 mol/L NaOH. Bacterial viability was determined by propidium iodide (PI) staining and quantified by flow cytometry (Accuri C6 Plus, BD Biosciences, San Jose, CA, USA). The inhibitory effect was expressed as the percentage of *H. pylori* death relative to the untreated control. Each experiment was carried out in three parallel replicates. Inhibition rate (%) was calculated according to the following formula:

Inhibition rate (%) = N_1_/N_0_ ∗ 100

where N_1_ represents the number of PI-positive (non-viable) cells, and N_0_ represents the total number of detected cells (PI-positive + PI-negative).

Organic acid profile: The concentrations of organic acids in the 24 h fermentation supernatant of *H. coagulans* BHE26 were determined by high-performance liquid chromatography (HPLC; LC-20AT, Shimadzu, Kyoto, Japan). Briefly, the strain was cultured in MRS broth at 37 °C for 24 h. Cultures were centrifuged at 10,000× *g* for 10 min using a high-speed refrigerated centrifuge (LYNX 6000, Thermo Fisher Scientific, Waltham, MA, USA). The CFS was filtered through a 0.22 μm membrane (Merck Millipore, Burlington, MA, USA). Chromatographic separation was performed using an Aminex HPX-87H column (300 × 7.8 mm, Bio-Rad Laboratories, Hercules, CA, USA) maintained at 30 °C, with phosphate buffer as the mobile phase at a flow rate of 1.0 mL/min. The detection was carried out at 215 nm. Lactic acid and acetic acid were used as standards.

### 2.4. Tolerance to Simulated Gastrointestinal Conditions

Preparation of gastric fluid: The pH of sodium chloride (0.3%, *w*/*v*) was adjusted to 3.0 with concentrated hydrochloric acid. After the addition of protease (0.35%, *w*/*v*), the solution was filtered with a 0.22 mm filter. The tolerance to artificial gastric fluid was assessed following the method of Zhang et al. with slight modifications [29]. Briefly, the suspension (0.5 mL, 10^8^ CFU/mL) of *H. coagulans* BHE26 was diluted in the artificial gastric fluid (4.5 mL) and incubated at 37 °C for 3 h. Then, astric fluid tolerance was assessed by mixing 0.5 mL of the suspension with 4.5 mL of simulated astric fluid, followed by static incubation at 37 °C for 24 h. The viable bacterial counts were determined using the pour plate method. Data are presented as:

Survival rate (%) = LgN_t_/LgN_0_

where N_t_ represents the number of surviving cells, and N_0_ denotes the number of initial cells.

Bile tolerance: The bile tolerance of *H. coagulans* BHE26 was evaluated as previously described by Angmo et al. with some modifications [30]. H. coagulans BHE26 (10%, *v*/*v*) was inoculated into GMRS broth containing 0% (*m*/*v*) and 1.0% (*m*/*v*) bile salts, respectively. After a reactivation step at 37 °C for 4 h, the culture (100 μL, 10^8^ CFU/mL) was serially diluted and spread onto agar plates. Although the physiological bile salt concentration in the human small intestine is approximately 0.3% [31], 1% was used to evaluate the maximum tolerance and robustness of the strain under extreme stress conditions. After a 24 h incubation at 37 °C, surviving colonies were enumerated using standard colony-forming unit (CFU) counts. All experiments were performed in three independent replicates to ensure reproducibility.

### 2.5. The Agglutination Activity of H. coagulans BHE26

#### 2.5.1. Hydrophobicity

The concentration of *H. coagulans* BHE26 cells was adjusted to 10^8^ CFU/mL with phosphate-buffered saline (PBS, 10 mmol/L, pH 7.4). Equal volumes of the *H. coagulans* BHE26 suspension (2 mL) and xylene (2 mL) were mixed and incubated at 37 °C for 1 h. *Lactobacillus plantarum* CN2018 was applied as the positive control. The OD_600_ was then measured. Hydrophobicity was calculated as follows:

Hydrophobicity (%) = (A_0_ − A_t_) ∗ 100%/A_0_.

where A_0_ represents the absorbance at 0 h, and A_t_ denotes the absorbance measured at time t (h) after the addition of xylene

#### 2.5.2. Auto-Aggregation

The auto-aggregation capacity of *H. coagulans* BHE26 was determined according to the method of Fonseca et al. with slight modifications [32]. The *H. coagulans* BHE26 cells were adjusted to 10^8^ CFU/mL with PBS (10 mmol/L, pH 7.4) and incubated statically at 37 °C for 48 h. The OD_600_ of the upper liquid was measured at 0, 2, 4, 6, 8, 16, 24, and 48 h. Auto-aggregation was calculated as follows:

Auto-aggregation (%) = (A_0_ − A_t_) ∗ 100%/A_0_

where A_0_ and A_t_ are the absorbance values of the upper liquid at 0 h and t h, respectively.

#### 2.5.3. Co-Aggregation

The concentrations of *H. pylori, H. coagulans* BHE26 and *Lactobacillus plantarum* CN2018 (positive control) were adjusted to 10^8^ CFU/mL with PBS (10 mmol/L, pH 7.4). The bacterial suspensions of *H. coagulans* BHE26 and *L. plantarum* CN2018 were each combined with the *H. pylori* in equal volumes and incubated statically at 37 °C for 48 h. The absorbance of the upper solution were measured at 600 nm at various times. Co-aggregation was calculated as follows:

Co-aggregation (%) = (A_0_ − A_mix_) ∗ 100%/A_0_

where A_0_ and A_mix_ refer to the OD_600_ of the upper solution at 0 h and at various incubation times, respectively.

### 2.6. Antibiotic Susceptibility and Safety Assessment

The antibiotic susceptibility of *H. coagulans* BHE26 was evaluated using the method of Huang et al. with slight modifications [33]. Briefly, *H. coagulans* BHE26 cells (1.0 × 10^8^ CFU/mL, 100 μL) was spread on GMRS agar plates. Antibiotic discs including penicillin (10 μg), gentamicin(10 μg), ampicillin (10 μg), erythromycin (15 μg), ciprofloxacin (5 μg), ceftriaxone (30 μg), lincomycin (2 μg), tetracycline (30 μg), chloramphenicol (30 μg), and trimethoprim-sulfamethoxazole (25 μg), were then placed on the surfaces of GMRS agar plates, respectively. After incubating the plates at 37 °C for 24 h, the inhibition zones were measured.

*H. coagulans* BHE26 was spot-inoculated onto a blood agar plate and incubated at 37 °C for 24 h. Hemolytic activity was classified as α-hemolysis (greenish discoloration surrounding the colonies), β-hemolysis (clear zones of lysis around the colonies), and γ-hemolysis (absence of any zone surrounding the colonies). *Staphylococcus aureus* served as the positive control.

### 2.7. Effect of H. coagulans BHE26 on H. pylori Infected Mice

#### 2.7.1. Animals and Treatments

A total of 108 male C57BL/6J mice (5 weeks old, 20–24 g) were purchased from Zhejiang Viton Lihua Laboratory Animal Technology Co., Ltd. (Jiaxing, China). All animal experiments were carried out according to the guidelines established by the Welfare and Ethics Review Committee of Zhengzhou University Laboratory Animal Center (approval number: ZZU-LAC20211015[15]). After one week of acclimatization, the mice were randomly allocated into two main experimental sets: the therapeutic intervention (n = 60) and preventive intervention (n = 48).

In the therapeutic experiment, mice were assigned to five groups (n = 12 per group): Control (Con), infection control (Hp_0), therapeutic control (Hp_GMRS), *H. pylori* treatment (Hp_E26), and supernatant treatment (Hp_E26_FS). The *H. pylori* infection model was established by oral gavage with 0.2 mL of *H. pylori* suspension (1 × 10^9^ CFU/mL) every other day for four doses. After infection, mice in the therapeutic groups received the following treatments for four weeks.

• Con group—gavaged daily with sterile water (0.2 mL/day);
• Hp_0 group—infected with *H. pylori* and gavaged daily with sterile water (0.2 mL/day);
• Hp_GMRS group—infected with *H. pylori* and gavaged daily with 0.2 mL of GMRS medium;
• Hp_E26 group—infected with *H. pylori* and treated daily with *H. coagulans* BHE26 suspension (5 × 10^8^ CFU/mL, 0.2 mL/day);
• Hp_E26_FS group—infected with *H. pylori* and treated daily with 24 h fermented supernatant of *H. coagulans* BHE26 (0.2 mL/day).

The dose, dosing frequency, oral gavage route, and treatment duration were selected to align with regimens commonly used in murine probiotic models of *H. pylori* infection.

In the preventive experiment, mice were divided into four groups (n = 12 per group): Control (Con), infection control (Hp_1), prevention group (E26_Hp), and *H. coagulans* BHE26-only group (E26). Treatments were arranged as follows:

• Con group—gavaged with sterile water throughout the experiment;
• Hp_1 group—gavaged with sterile water for 3 weeks prior to *H. pylori* infection and continued during infection;
• E26_Hp group—gavaged with *H. coagulans* BHE26 (5 × 10^8^ CFU/mL, 0.2 mL/day) for 3 weeks before *H. pylori* challenge and continuously treated during infection;
• E26 group—gavaged with *H. coagulans* BHE26 (5 × 10^8^ CFU/mL, 0.2 mL/day) for 4 weeks without *H. pylori* challenge.

After overnight fasting, blood was collected from the retro-orbital plexus. Serum was separated by centrifugation at 3000× *g* for 15 min at 4 °C and stored at −80 °C. Mice were then euthanized by cervical dislocation, and the stomachs were removed aseptically. Each stomach was rinsed with sterile PBS (0.01 mol/L, pH 7.4) to remove residual contents, and tissues from the pyloric region were collected. The samples were divided into three parts: one for urease activity assay after homogenization in sterile saline (1:9, *w*/*v*), one snap-frozen in liquid nitrogen for 16S rRNA sequencing, and one fixed in 4% neutral buffered formalin for histological examination.

#### 2.7.2. Urease Activity

Gastric urease activity was determined using a modified phenol-red rapid urease test [34]. Briefly, 50 µL of gastric tissue homogenate was mixed with 150 µL of urease and incubated under microaerophilic conditions for 24 h at 37 °C. A color change from yellow to purple indicated positive urease activity.

#### 2.7.3. Histopathological Analysis of Gastric Tissue and Measurement of Anti-Bacterial-IgG Antibodies in Mouse Serum

Gastric tissues were fixed in 4% neutral-buffered formalin, paraffin-embedded, and sectioned at 4–6 μm. Sections were stained with hematoxylin–eosin (H&E) for histopathological assessment and with Warthin–Starry silver stain to visualize *H. pylori*. Slides were examined under a light microscope (Leica, Wetzlar, Germany). Serum anti-bacterial IgG was measured by ELISA (Cusabio, Wuhan, China) according to the manufacturer’s instructions. Absorbance was measured at 450 nm.

#### 2.7.4. Analysis of Gastric Tissue Microbiota

Biological information was analyzed by targeting the V4 and V5 regions of the mouse gastric tissue using 16S rDNA amplicon sequencing. PCR amplification was performed with the forward primer (GTGCCAGCMGCCGCGGTAA) and reverse primer (CCGTCAATTCCTTTGAGTTT). Amplicon sequence variant (ASV) noise reduction was applied to process the sequencing data, enhancing the accuracy of the analysis [35]. The validated data were subsequently used for taxonomic annotation and abundance profiling. Sequencing depth and coverage were assessed using rarefaction curves and Good’s coverage index, with most samples exceeding 97% [36]. To investigate variations in community structure across samples, beta-diversity metrics and Linear Discriminant Analysis Effect Size (LEfSe) analysis were employed [37].

### 2.8. Statistical Analysis

Data analysis was conducted using one-way analysis of variance (ANOVA) with Tukey’s honestly significant difference (HSD) test, considering a significance level of *p* < 0.05, using SPSS 26.0. The results are expressed as means ± standard deviations.

## 3. Results and Discussion

### 3.1. Anti-Bacterial Activity of H. coagulans BHE26

The growth inhibition assay against *H. pylori* serves as a key and direct indicator for the screening of probiotics [38]. The fermentation broths and supernatants of *Lactobacillus plantarum* CN2018 exhibited distinct inhibitory effects against *H. pylori*, with inhibition zone diameters ranging from 12.44 mm to 14.29 mm (Table 1). No inhibition zones were observed for the bacterial suspension of *H. coagulans* BHE26, similar to the negative control (GMRS ), suggesting that the antibacterial activity may be attributed to extracellular metabolites. The fermentation broth and cell-free supernatant of *H. coagulans* BHE26 produced inhibition zones of 13.19 ± 0.46 mm and 13.76 ± 0.22 mm, respectively (Table 1), indicating that the antibacterial activity is primarily mediated by extracellular metabolites. In agar diffusion assays, inhibition zones in the range of approximately 10–20 mm are generally considered to reflect moderate but biologically relevant antibacterial activity [39]. The inhibition zone diameters observed in the present study (approximately 12–14 mm) are comparable to those reported for other probiotic strains against *H. pylori*, supporting the biological significance of the antagonistic effects [40,41].

The absence of inhibition by bacterial suspensions, together with the clear activity of fermentation broths and cell-free supernatants, indicates that secreted antimicrobial compounds mediate the antagonistic effects. Probiotic strains produce extracellular metabolites, including organic acids, antimicrobial peptides, and bacteriocins, which inhibit *H. pylori* growth and adhesion in vivo. Lyophilized cell-free supernatants of *Limosilactobacillus fermentum* T0701, containing these secreted compounds, strongly suppressed *H. pylori* growth and reduced bacterial adhesion to epithelial cells [41]. These extracellular effects correspond with the observed reduction in gastric colonization in vivo.

### 3.2. Analysis of Antimicrobial Components in the CFC

**Figure 1:**
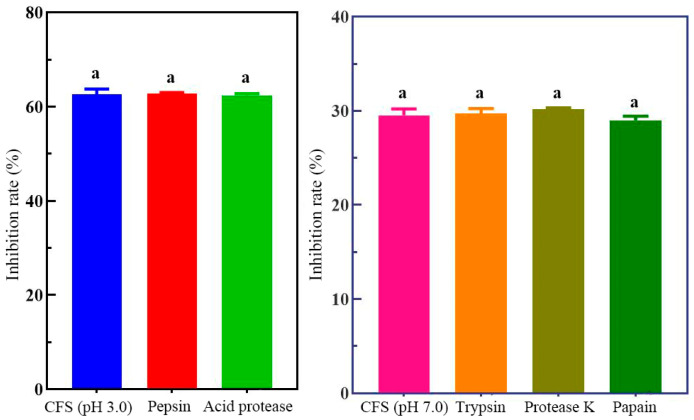
Effect of protease treatment on the antibacterial activity of *H. coagulans* BHE26 CFS against *H. pylori*. The CFS was treated with acidic proteases (pepsin and acid protease) or neutral proteases (trypsin, proteinase K, and papain) prior to incubation with *H. pylori*. The inhibitory effect was expressed as the percentage of *H. pylori* death, calculated relative to the untreated control. The same letter indicates no significant difference (*p* < 0.05).

**Figure 2:**
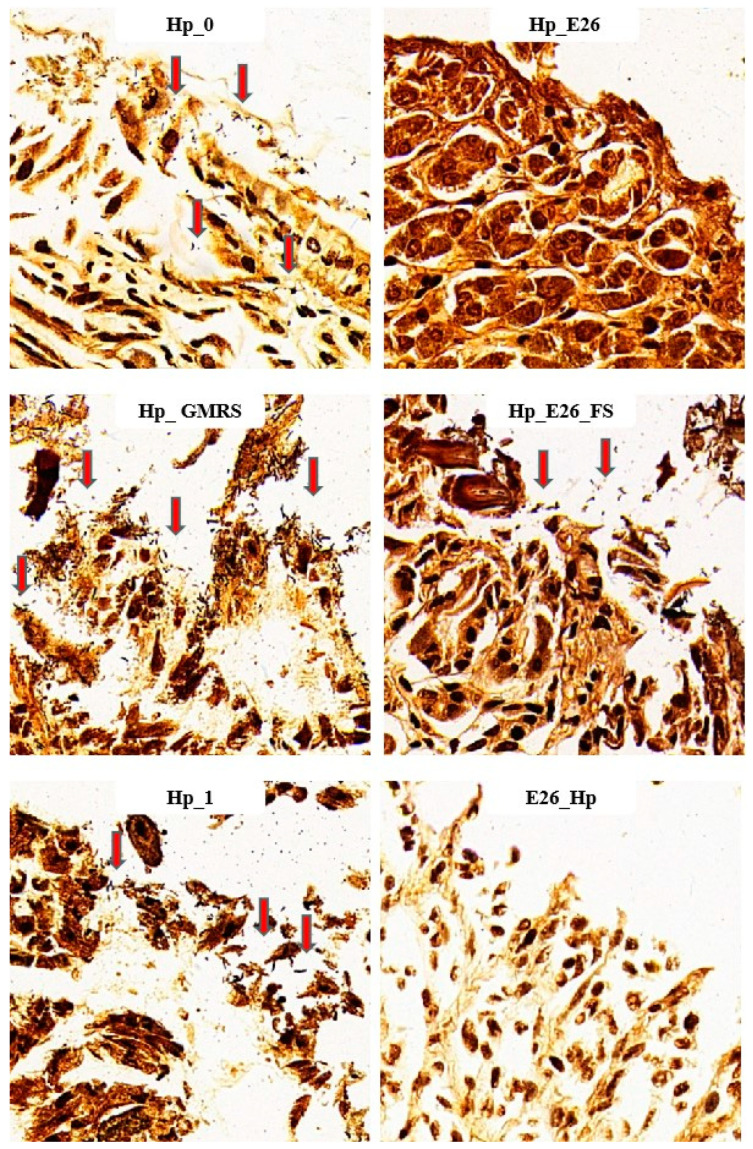
W-S silver staining of gastric tissue sections showing *H. pylori* (red arrows) at 400× magnification. Hp_0, Hp_E26, Hp_GMRS, Hp_E26_FS, Hp_1, and E26_Hp denote infection control in the therapeutic experiment, *H. pylori* treatment, therapeutic control, supernatant treatment, infection control in the preventive experiment and prevention group, respectively.

Incubation with acidic proteases (pepsin and acid protease) or neutral proteases (trypsin, proteinase K, and papain) resulted in no significant changes in *H. pylori* viability compared with the untreated control (*p* > 0.05) (Figure 1). These results suggest that the inhibitory activity of the cell-free supernatant is not protein-based. HPLC analysis showed that lactic acid was the predominant organic acid in the supernatant of *H. coagulans* BHE26 after 24 h of fermentation (35.8 mmol/L), followed by acetic acid (31.7 mmol/L). The dominance of lactic acid, together with the marked reduction in inhibitory activity after pH neutralization, supports its major role in suppressing *H. pylori* growth (Figure 2). Lactic acid has been reported to enhance antibacterial effects by suppressing *H. pylori* urease activity and increasing bacterial membrane permeability [42]. Acetic acid, although present at lower concentrations, may contribute synergistically to inhibition.

### 3.3. Tolerance to Simulated Gastrointestinal Conditions

The ability of probiotics to survive gastrointestinal transit is a key criterion for exerting beneficial effects on the host. Low pH and bile salts are the initial physiological challenges faced by probiotic strains during gastrointestinal passage [43]. After 3 h of incubation in artificial gastric fluid at pH 3.0, *H. coagulans BHE26* exhibited a survival rate of 69.3%, indicating effective tolerance to acidic conditions. *Lactobacillus plantarum* CN2018 (positive control) showed a survival rate of 79.1%. Bile salt tolerance is critical for probiotics in vivo. Bile salts can damage the cell structure, with the extent of damage increasing at higher concentrations. *H. coagulans BHE26* exhibited a survival rate of 85.8% after 4 h of incubation in MRS broth with 1% bile salt, indicating its strong tolerance to high bile salt concentrations. Although the physiological bile salt concentration in the human small intestine is approximately 0.3%, 1% was intentionally used to evaluate the strain’s maximum tolerance and robustness under extreme stress [31]. Under the same conditions, *L. plantarum* CN2018 exhibited a survival rate of 50.1%. Hyronimus et al. found that *Bacillus coagulans* and *B. racemilacticus* were unable to survive under such harsh conditions [44]. In contrast, Nithya and Halami reported that *B. subtilis* Bn1 exhibited tolerance to 0.3% ox-bile, whereas *B. flexus* Hk1 was susceptible [45]. Overall, these results indicate that *H. coagulans* BHE26 can withstand acidic pH and high bile salt stress, supporting its potential suitability as a probiotic capable of surviving gastrointestinal transit.

### 3.4. The Agglutination Activity of H. coagulans BHE26

*H. coagulans* BHE26 exhibited a hydrophobicity of 45.8%, which was significantly higher than that of the positive control *Lactobacillus plantarum* CN2018 (21.9%, *p* < 0.05). However, hydrophobicity alone is not considered a reliable predictor of epithelial adhesion in vivo. Hydrophobicity, auto-aggregation, and co-aggregation represent strain-specific physicochemical traits which are often associated with adhesion potential in vitro [46]. These relationships vary markedly among strains and experimental conditions [46,47]. *H. coagulans* BHE26 displayed a high auto-aggregation percentage of 68% after 24 h, exceeding CN2018 (61%, *p* < 0.05). In addition, it demonstrated strong co-aggregation with *H. pylori*, reaching 64.7% and 79.4% at 24 and 48 h, respectively, which were significantly higher than the corresponding values observed for *L*. *plantarum* CN2018 (62.1% and 70.2%, *p* < 0.05).

Previous studies have reported that specific co-aggregation between probiotics and *H. pylori* is associated with reduced pathogen adhesion to epithelial surfaces in vitro [40,48]. Co-aggregation can promote physical association between probiotics and *H. pylori.* Administration of *Lactobacillus reuteri* DSM 17648 has been linked to reduced gastric *H. pylori* burden in vivo [49]. Moreover, clinical trials have demonstrated beneficial adjunctive effects of *L. reuteri* DSM17648, including reduced *H. pylori* load and improved treatment tolerability [50]. These findings are consistent with passive removal through aggregate formation as a plausible, strain-specific mechanism, while competitive exclusion and other complementary effects are also likely to contribute.

### 3.5. Antibiotic Susceptibility Testing and Safety Evaluation

*H. coagulans* BHE26 was resistant to ceftriaxone but sensitive to penicillin, ampicillin, erythromycin, gentamicin, ciprofloxacin, ceftriaxone, lincomycin, tetracycline and chloramphenicol (Table 2). Certain resistance phenotypes in probiotic genera, including *Lactobacillus, Bifidobacterium*, and *Bacillus*, are intrinsic and non-transferable and therefore do not pose an inherent safety concern in the absence of mobile genetic elements [51]. According to EFSA, only acquired antimicrobial resistance genes carried on mobile genetic elements are considered a relevant safety concern, whereas intrinsic, species-associated resistance is generally not considered a safety concern in the absence of evidence for transferability [51,52]. *H. coagulans* BHE26 exhibited resistance to ceftriaxone but was susceptible to multiple clinically important antibiotics. The results are consistent with other *Bacillus* strains showing predominantly intrinsic resistance patterns and broad susceptibility profiles, as evidenced in *Bacillus velezensis* where potential resistance genes are chromosomally encoded without mobile elements and with broad antibiotic sensitivity [53]. Thus, *H. coagulans* BHE26’s antibiotic susceptibility profile aligns with current safety evaluation frameworks. To further ensure the safety of the *H. coagulans* BHE26, a hemolysis assay was performed. *H. coagulans* BHE26 strain exhibited γ-hemolytic activity (Table 3), indicating it is non-hemolytic and safe.

### 3.6. Effect of H. coagulans BHE26 on H. pylori-Infected Mice

#### 3.6.1. Urease Activity and W–S Silver Staining Analyses

As shown in Table 4, both the blank control (Con) and the *H. coagulans* BHE26-only group (E26) were urease-negative, confirming the absence of endogenous *H. pylori* infection. In the *H. pylori* model groups, urease positivity ranged from 80% to 100%, whereas daily oral administration of *H. coagulans* BHE26 markedly reduced urease-positive rates to 30–40% in both the bacterial suspension (Hp_E26) and fermented supernatant (Hp_E26_FS) groups. Notably, preventive pretreatment with *H. coagulans* BHE26 (E26_Hp) resulted in the lowest urease positivity of 17%. W-S silver staining further corroborated these findings (Figure 2). Abundant brown-black, rod- and spiral-shaped *H. pylori* cells were observed on the gastric mucosal surface and within glandular lumens in infected control groups, whereas gastric colonization was substantially reduced following *H. coagulans* BHE26 treatment. The positive staining rates decreased from 80 to 100% in infected controls to 40% (Hp_E26), 50% (Hp_E26_FS), and 25% (E26_Hp), demonstrating a marked suppression of *H. pylori* colonization. Probiotics have been shown to reduce urease activity and colonization through mechanisms such as growth inhibition and competitive adhesion, while complete bactericidal elimination by probiotics alone is rarely observed [40,54,55]. The dosing regimen, including daily oral gavage and a multi-week treatment period, was consistent with that used in previous studies investigating probiotic interventions against *H. pylori*. For example, *Lactobacillus plantarum* ZFM4 and *Lacticaseibacillus casei* T1 have been administered by daily oral gavage at comparable doses (10^8^–10^9^ CFU per dose) for multiple weeks, resulting in reduced gastric colonization and urease activity without complete eradication of *H. pylori* [56,57]. The Hp_GMRS group, which received GMRS medium alone as a vehicle control, showed no significant changes in urease activity and bacterial colonization. This indicates that the observed effects were specifically attributable to *H. coagulans* BHE26 and its fermentation-derived products. These results demonstrate that *H. coagulans* BHE26 effectively suppresses *H. pylori* colonization and urease activity in vivo.

#### 3.6.2. Measurement of Anti-Bacterial-IgG Antibodies in Mouse Serum

**Figure 3:**
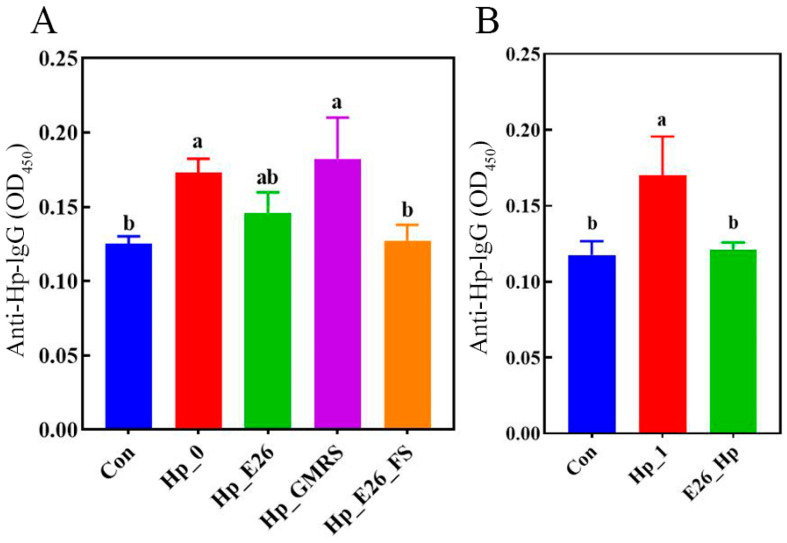
Levels of anti-bacterial IgG antibodies in mouse serum (**A**) therapeutic intervention group; (**B**) prophylactic intervention group. Different letters (a, b) indicate significant differences from each other (*p* < 0.05). Statistical analysis was performed using one-way ANOVA with Tukey’s post hoc test.

In both treatment and prevention groups, serum anti-bacterial IgG levels were significantly higher in the infection controls (Hp_0 and Hp_1) than in the blank control (Con) (Figure 3, *p* < 0.05). Following probiotic intervention, antibody levels generally declined, except in the Hp_GMRS group, where no significant difference was observed. Notably, the Hp_E26_FS group exhibited a significant reduction in anti-bacterial IgG levels (*p* < 0.05). These results demonstrate that *H. pylori* infection was associated with a marked increase in serum antibacterial IgG levels, whereas treatment with *H. coagulans* BHE26 significantly attenuated this response. This decrease is likely attributable to reduced antigenic stimulation resulting from diminished gastric colonization and alleviated mucosal inflammation, rather than to a direct modulation of humoral immunity [58,59]. This observation aligns with previous study that administration of *Lactobacillus* strains to *H. pylori*-infected mice reduces gastric bacterial load and concurrently lowers serum antibody levels [60].

#### 3.6.3. *H. coagulans* BHE26 Inhibits *H. pylori* Infection In Vivo

**Figure 4:**
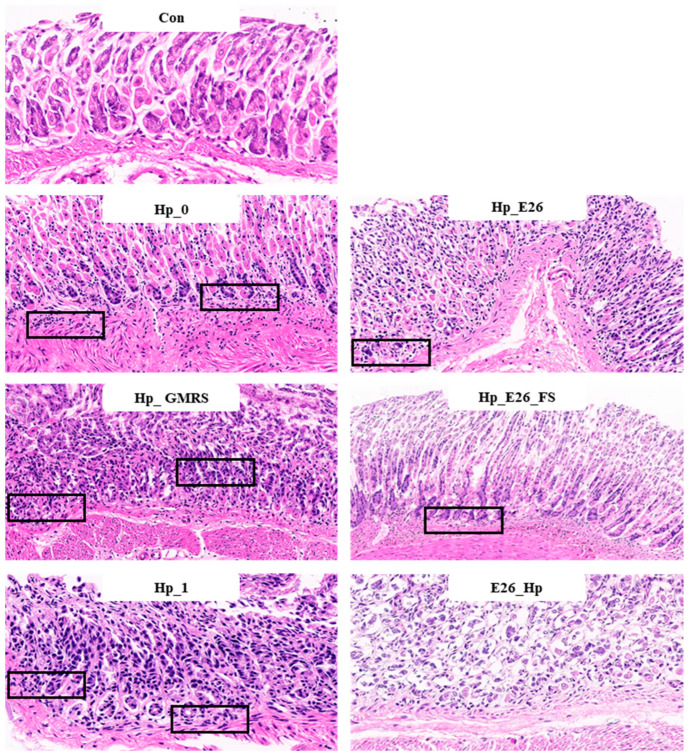
Hematoxylin and eosin (H&E) staining of gastric tissue sections at 200× magnification. Black boxes indicate inflammatory cell infiltration. Con, Hp_0, Hp_E26, Hp_GMRS, Hp_E26_FS, Hp_1, and E26_Hp denote blank control group, infection control in the therapeutic experiment, *H. pylori* treatment, therapeutic control, supernatant treatment, infection control in the preventive experiment and prevention group, respectively.

H&E staining revealed that *H. pylori* infection caused significant disruption of the gastric mucosal architecture, accompanied by extensive infiltration of immune cells (Figure 4). In contrast, mice treated with *H. coagulans* BHE26 showed preserved gastric structures and markedly reduced inflammatory infiltration. Mild inflammation observed in the control group was attributed to mechanical intragastric administration. Both post-infection (Hp_E26) and pre-infection (E26_Hp) treatments with *H. coagulans* BHE26 significantly alleviated mucosal inflammation compared to their respective controls (Hp_0 and Hp_1), whereas the GMRS treatment showed no improvement (Figure 4). The fermentation supernatant (Hp_E26_FS) also decreased inflammatory cell infiltration, suggesting that bioactive metabolites from *H. coagulans* BHE26 contributed to its anti-inflammatory effects (Figure 4). These findings indicate that both the bacterial suspension and fermentation supernatant of *H. coagulans* BHE26 exert protective effects against *H. pylori*-induced gastric mucosal injury. The anti-inflammatory effect appears to be mediated by reduced *H. pylori* colonization and urease activity, and enhanced mucosal immune defense [61].

### 3.7. Bioinformatic Analysis of Gastric Microbiota

#### 3.7.1. Beta Diversity Analysis

**Figure 5:**
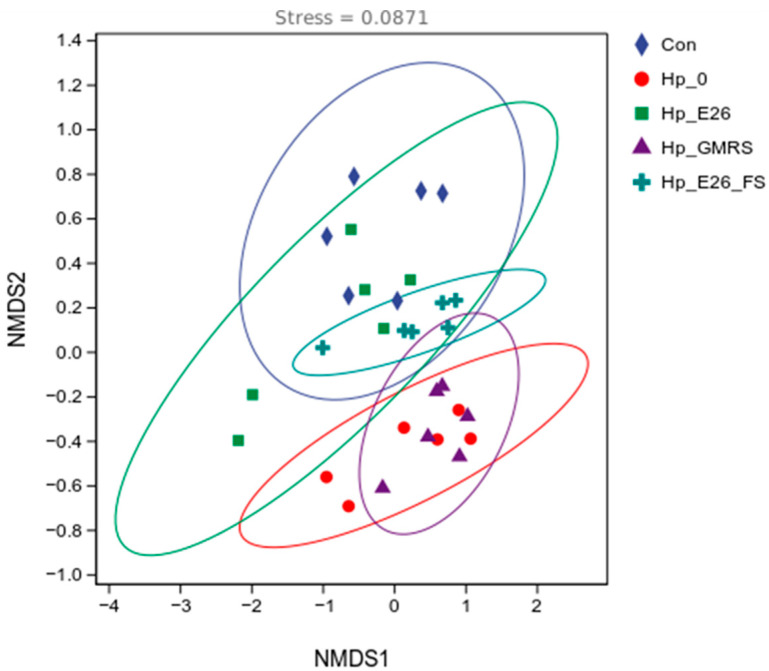
Beta-diversity analysis. Con, Hp_0, Hp_E26, Hp_GMRS, Hp_E26_FS, denote blank control group, infection control in the therapeutic experiment, *H. pylori* treatment, therapeutic control, and supernatant treatment, respectively.

Non-metric multidimensional scaling (NMDS) based on Jaccard distances revealed clear separation of gastric microbiota among experimental groups (stress = 0.0871; Figure 5). The Hp_0 group clustered distinctly from controls, indicating substantial *H. pylori*-induced dysbiosis. In contrast, the Hp_E26 group overlapped considerably with controls, suggesting that bacterial suspension intervention effectively attenuated microbial composition toward a healthy state. The Hp_E26_FS and Hp_GMRS groups formed intermediate clusters, with minimal overlap, highlighting differential modulatory effects of bacterial supernatant versus GMRS treatment. Adonis analysis confirmed these observations, showing significant differences between control and Hp_0 groups (*p* < 0.01) and between Hp_E26 and Hp_0 (*p* < 0.05), while no significant difference was observed between Hp_E26 and controls (*p* > 0.05). The Hp_E26_FS and Hp_GMRS groups also differed significantly (*p* < 0.01), reinforcing that distinct intervention modalities yield divergent effects on the gastric microbial community.

#### 3.7.2. Phylum Level and Genus Level Species Analysis

**Figure 6:**
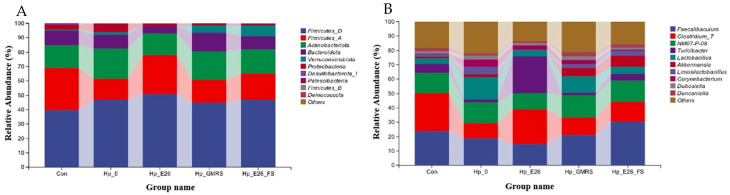
Histogram of relative abundance of gastric microbiota at phylum level (**A**) and genus level (**B**). Con, Hp_0, Hp_E26, Hp_GMRS, Hp_E26_FS, denote blank control group, infection control in the therapeutic experiment, *H. pylori* treatment, therapeutic control, and supernatant treatment, respectively.

At the phylum level, the dominant bacterial taxa were similar across all groups (Figure 6A), including *Firmicutes*, *Actinobacteriota*, *Bacteroidota*, *Verrucomicrobiota*, and *Proteobacteria*, although their relative abundances differed. Compared with the control group, *H. pylori* infection (Hp_0) resulted in a decrease in *Firmicutes* and *Bacteroidota* and an increase in *Actinobacteriota*. At the genus level, the major bacterial genera included *Faecalibaculum*, *Clostridium_T*, *Lactobacillus*, *Corynebacterium*, *Akkermansia*, and *Dubosiella* (Figure 6B). Administration of *H. coagulans* BHE26 to *H. pylori*-infected mice enriched gastric *Limosilactobacillus* and *Dubosiella*, indicating functional microbiota modulation under dysbiosis rather than contamination [62]. Probiotics may suppress *H. pylori* via competitive exclusion, modulation of microbial metabolites, and attenuation of mucosal inflammation [62,63]. These microbial shifts coincided with significant reductions in gastric *H. pylori* colonization and serum anti-*H. pylori* IgG levels, supporting a contributory role of microbiota remodeling in pathogen suppression.

#### 3.7.3. Linear Discriminant Analysis Effect Size (LEfSe) Analysis

**Figure 7:**
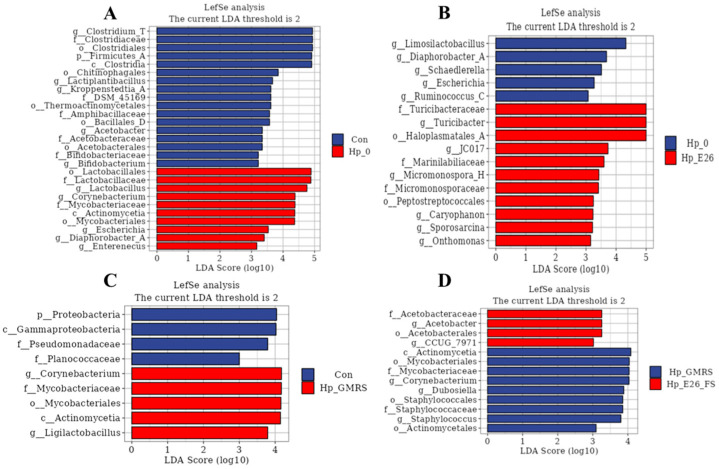
LEfSe multi-level taxonomic analysis of gastric microbiota. (**A**) Differentially abundant taxa between the Con and Hp-0 groups. (**B**) Differentially abundant taxa between the Hp-0 and Hp-E26 groups. (**C**) Differentially abundant taxa between the Con and Hp-GMRS groups. (**D**) Differentially abundant taxa between the Hp-GMRS and Hp-E26-FS groups. Con, Hp-0, Hp-E26, Hp-GMRS, Hp-E26-FS denote blank control group, infection control group, therapeutic group 1, therapeutic control group, therapeutic group 2, respectively.

LEfSe analysis revealed that *H. pylori* infection significantly reshaped the gastric microbiota in mice. Compared with the control group, the Hp_0 group showed enrichment of *Lactobacillales*, *Corynebacterium*, *Actinobacteria*, and *Mycobacteriales*, along with a reduction in *Clostridia* and *Firmicutes*_A (LDA score > 4; Figure 7A). The Hp_E26 group was characterized by increased *Turicibacter* and *Haloplasmatales*_A, while *Lactobacillus* abundance decreased relative to Hp_0 (LDA score > 4; Figure 7B). Reduced *Lactobacillus* abundance has been frequently reported in *H. pylori*-infected hosts, as specific *Lactobacillus* strains can inhibit *H. pylori* growth or adhesion through organic acid production and competitive exclusion [64,65]. An increased abundance of *Turicibacter* was also detected in Hp_E26 group (Figure 7B). *Turicibacter* strains are associated with host bile acid profiles and lipid metabolism [66]. The enrichment of *Turicibacter* observed in this study is interpreted as a community-level shift reflecting altered host or environmental conditions. In addition, the Hp_E26_FS group exhibited lower levels of *Actinobacteria*, *Mycobacteriales*, and *Corynebacterium* than the Hp_GMRS group (LDA score > 4; Figure 7D). *H. pylori* infection induced gastric dysbiosis by disrupting microbial balance, whereas treatment with the bacterial suspension or supernatant of *H. coagulans* BHE26 effectively alleviated these alterations, restoring the microbiota toward a composition resembling that of healthy controls. Probiotic interventions can remodel the gastric microbiota and counteract *H. pylori*-mediated dysbiosis [67].

## 4. Conclusions

This study aimed to systematically evaluate the potential of *H. coagulans* BHE26 against *H. pylori* in vitro and in vivo. *H. coagulans* BHE26 directly inhibited *H. pylori* growth in vitro. It expressed remarkable hydrophobicity, auto-aggregation and co-aggregation ability. In the *H. pylori*-infected mouse model, administration of *H. coagulans* BHE26 significantly reduced *H. pylori* colonization and urease activity, as well as serum *H. pylori*-specific IgG levels. Moreover, this strain effectively modulated the composition of the gastric microbiota toward a healthier profile, suggesting its role in supporting gastric microbial homeostasis. These findings indicate that *H. coagulans* BHE26 exhibits promising probiotic potential and may be considered for future application in functional food industry.

## Figures and Tables

**Table 1:** Effects of fermentation broth and supernatant of *H. coagulans* BHE26 on the diameter (mm) of the inhibition zone of *H. pylori* *.

Strains	Fermentation Broth (mm)	Fermentation Supernatant (mm)
Negative control (GMRS)	6.00 ± 0.00 ^c^	6.00 ± 0.00 ^c^
*Lactobacillus plantarum* CN2018	14.29 ± 0.23 ^a^	12.44 ± 0.59 ^b^
*H. coagulans* BHE26	13.19 ± 0.46 ^b^	13.76 ± 0.22 ^a^

* The data are shown as the means ± standard deviations. Different letters (a–c) indicate significant differences from each other (*p* < 0.05). Statistical analysis was performed using one-way ANOVA with Tukey’s post hoc test.

**Table 2:** Antibiotic sensitivity of *H. coagulans* BHE26.

Types of Antibiotic	Disc Content (µg)	Diameter of Inhibition Zone (mm)			Susceptibility
		Resistant (R)	Intermediate (I)	Susceptible (S)	*H. coagulans* BHE26
Penicillin	10	≤14	-	≥18	S
Ampicillin	10	≤13	14~16	≥17	S
Erythromycin	15	≤13	14~22	≥23	S
Gentamicin	10	≤12	13~14	≥15	S
Ciprofloxacin	5	≤12	13~18	≥18	S
Ceftriaxone	30	≤11	12~16	≥17	R
Lincomycin	2	≤10	11~15	≥16	S
Tetracycline	30	≤10	11~15	≥16	S
Chloramphenicol	30	≤10	11~15	≥16	S
Trimethoprim-Sulfamethoxazole	25	≤10	11~15	≥16	S

Values are the mean of three experiments, each with duplicate samples; Resistant (R), I—Intermediate, S—susceptible.

**Table 3:** Hemolytic activity of *H. coagulans* BHE26.

Strains	Hemolytic Activity
*H. coagulans* BHE26	γ
*S. aureus*	β

β: β hemolysis; γ: γ hemolysis.

**Table 4:** Urease activity and W-S silver staining results of gastric tissue in mice.

Group	Urease Activity (Positive/Total)	W-S Silver Staining (Positive/Total)	Group	Urease Activity (Positive/Total)	W-S Silver Staining (Positive/Total)
Con	0/5	0/5	Con	0/6	0/6
Hp_0	8/10	9/10	Hp_1	6/6	6/6
Hp_E26	4/10	4/10	E26_Hp	2/12	3/12
Hp_GMRS	8/10	8/10	E26	0/12	-
Hp_E26_FS	3/10	5/10	-	-	-

-: Not tested.

## Data Availability

The original contributions presented in the study are included in the article, further inquiries can be directed to the corresponding authors.
